# Supplementary material for: Ataxin-3 Links NOD2 and TLR2 Mediated Innate Immune Sensing and Metabolism in Myeloid Cells
Source: Front Immunol. 2019 Jul 19;10:1495. doi: 10.3389/fimmu.2019.01495 (PMC6659470; doi:10.3389/fimmu.2019.01495)
Supplement: Supplementary file 1 [file Data_Sheet_1.docx]

**Supplementary information**

Supplementary Figure 1 (S1)

SYBR Green based qPCR of a mitochondrial DNA fragment (mitochondrially encoded tRNA leucine 1) was performed, and the relative mtDNA copy number calculated by normalising to simultaneous qPCR of a nuclear DNA fragment (β2 microglobulin). n=2.

Supplementary Figure 2 (S2)

THP-1 cell viability in ataxin-3 and control shRNA THP-1 cells infected for 1-6hr with *Salmonella enterica* serovar Typhimurium. n=3.

**Supplementary Table 1**

Proteins found to be differentially phosphorylated in phosphoproteomic analysis of human moDCs following MDP+PAM_3_CSK_4_ stimulation

| **Protein (Gene)** | **Log2 fold change** |
| --- | --- |
| Ubiquitin-like modifier-activating enzyme 6 (UBA6) | 5.36 |
| Coagulation factor X (F10) | 4.70 |
| GA-binding protein alpha chain (GABPA) | 4.57 |
| DnaJ homolog subfamily B member 1 (DNAJB1) | 4.02 |
| Ubiquitin thioesterase otulin (OTULIN) | 3.88 |
| THAP domain-containing protein 11 | 3.36 |
| ADP-ribosylation factor-binding protein GGA1 (GGA1) | 3.12 |
| TNF receptor-associated factor 6 (TRAF6) | 3.03 |
| BRCA1-associated ATM activator 1 (BRAT1) | 2.95 |
| Trifunctional purine biosynthetic protein adenosine-3 (GART) | 2.91 |
| Dual specificity mitogen-activated protein kinase kinase 6 (MAP2K6) | 2.88 |
| Ataxin-3 (ATXN3) | 2.86 |
| Myeloma-overexpressed gene 2 protein (MYEOV2) | 2.74 |
| Glutamine-dependent NAD(+) synthetase (NADSYN1) | 2.72 |
| Zinc finger MIZ domain-containing protein 1 (ZMIZ1) | 2.71 |
| Bromodomain-containing protein 4 (BRD4) | 2.68 |
| Histone deacetylase 6 (HDAC6) | 2.63 |
| Leucine-rich repeat-containing protein 41 (LRRC41) | 2.58 |
| Vacuolar-sorting protein SNF8 (SNF8) | 2.48 |
| Vacuolar protein sorting-associated protein VTA1 homolog (VTA1) | 2.37 |
| Beta-adrenergic receptor kinase 1 (ADRBK1) | 2.34 |
| Target of Myb protein 1 (TOM1) | 2.34 |
| Endophilin-B1 (SH3GLB1) | 2.27 |
| Translocon-associated protein subunit beta (SSR2) | 2.21 |
| Protein S100-A6 (S100A6) | 2.19 |
| AN1-type zinc finger protein 5 (ZFAND5) | 2.18 |
| Short coiled-coil protein (SCOC) | 2.18 |
| Signal transducer and activator of transcription 6 (STAT6) | 2.16 |
| Signal transducer and activator of transcription 1-alpha/beta (STAT1) | 2.13 |
| 26S proteasome non-ATPase regulatory subunit 4 (PSMD4) | 2.10 |
| Coagulation factor IX (F9) | 2.07 |
| ADP-ribosylation factor-binding protein GGA2 (GGA2) | 2.06 |
| Syntenin-1 (SDCBP) | 2.04 |
| Dual specificity mitogen-activated protein kinase kinase 3 (MAP2K3) | 2.03 |
| Vitronectin (VTN) | 2.01 |
| Protein NEDD1 (NEDD1) | 2.00 |
| Mitogen-activated protein kinase 14 (MAPK14) | 1.97 |
| 2'-deoxynucleoside 5'-phosphate N-hydrolase 1 (DNPH1) | 1.95 |
| Proteasome inhibitor PI31 subunit (PSMF1) | 1.93 |
| CTD small phosphatase-like protein 2 (CTDSPL2) | 1.90 |
| Anamorsin (CIAPIN1) | 1.90 |
| Proteasomal ubiquitin receptor ADRM1 (ADRM1) | 1.89 |
| Tristetraprolin (ZFP36) | 1.89 |
| NLR family CARD domain-containing protein 4 (NLRC4) | 1.87 |
| Heat shock protein beta-1 (HSPB1) | 1.87 |
| Coronin-7 (CORO7) | 1.85 |
| Hyaluronan-binding protein 2 (HABP20 | 1.84 |
| Phosphatidylinositol-binding clathrin assembly protein (PICALM) | 1.79 |
| Receptor-interacting serine/threonine-protein kinase 1 (RIPK1) | 1.78 |
| Nipped-B-like protein (NIPBL) | 1.74 |
| Exportin-1 (XPO1) | 1.73 |
| Host cell factor 1 (HCFC1) | 1.70 |
| AN1-type zinc finger protein 6 (ZFAND6) | 1.69 |
| Golgi reassembly-stacking protein 2 (GORASP2) | 1.69 |
| Protein FAM161B (FAM161B) | 1.65 |
| Tensin-like C1 domain-containing phosphatase (TENC1) | 1.63 |
| Stathmin (STMN1) | 1.62 |
| ATP-dependent RNA helicase DHX29 (DHX29) | 1.61 |
| Vacuolar protein-sorting-associated protein 25 (VP25) | 1.61 |
| Dual specificity mitogen-activated protein kinase kinase 4 (MAP2K4) | 1.59 |
| TRAF family member-associated NF-kappa-B activator (TANK) | 1.59 |
| Ubiquitin carboxyl-terminal hydrolase 25 (USP25) | 1.56 |
| Ankyrin repeat domain-containing protein 13A (ANKRD13A) | 1.55 |
| Retinoic acid receptor RXR-alpha (RXRA) | 1.54 |
| Toll-interacting protein (TOLLIP) | 1.53 |
| Spartin (SPG20) | 1.53 |
| SEC23-interacting protein (SEC23IP) | 1.53 |
| Probable helicase senataxin (SETX) | 1.53 |
| Sorting nexin-12 (SNX12) | 1.52 |
| CREB-binding protein (CREBBP) | 1.50 |

**Supplementary Table 2**

Proteins found to be differentially ubiquitinated in TUBES analysis comparing control and ataxin-3 depleted THP-1 cells

| **Protein (Gene)** | **Log2 fold change** |
| --- | --- |
| E3 ubiquitin-protein ligase pellino homolog 1 (PEL1) | 5.38 |
| Isoform 2 of Integral membrane protein 2B (ITM2) | 3.79 |
| Mitogen-activated protein kinase kinase kinase kinase 1 (MAP4K1) | 3.63 |
| Lysosomal alpha-glucosidase (GAA) | 3.52 |
| Lys-63-specific deubiquitinase BRCC36 (BRCC3) | 3.36 |
| Signal transducer and activator of transcription (STAT3) | 2.68 |
| 39S ribosomal protein L23, mitochondrial (MRPL23) | 2.43 |
| 39S ribosomal protein L14, mitochondrial (MRPL14) | 2.33 |
| ATP-dependent DNA helicase Q1 (RECQL) | 2.22 |
| Rootletin (CROCC) | 2.15 |
| Isoform Beta-1 of DNA topoisomerase 2-beta (TOP2B) | 2.11 |
| Host cell factor 1 (HCFC1) | 2.11 |
| Homeodomain-interacting protein kinase 1 (HIPK1) | 2.10 |
| Sodium-dependent lysophosphatidylcholine symporter 1 (MFSD2A) | 2.06 |
| ATP-dependent RNA helicase A (DHX9) | 1.96 |
| Isoform 2 of E3 SUMO-protein ligase ZNF451 (ZNF451) | 1.95 |
| Protein transport protein Sec23B (SEC23B) | 1.88 |
| Pleckstrin (PLEK) | 1.88 |
| 1-acyl-sn-glycerol-3-phosphate acyltransferase epsilon (AGPAT5) | 1.80 |
| Isoform 2 of Cyclin-dependent kinase 2 (CDK2) | 1.79 |
| Uncharacterized protein | 1.74 |
| Isoform 3 of Hypoxia-inducible factor 1-alpha (HIF1A) | 1.71 |
| Isoform 2 of Xaa-Pro aminopeptidase 1 (XNPEP1) | 1.70 |
| Folate transporter 1 (SLC19A1) | 1.69 |
| Syndecan-2 (SDC2) | 1.66 |
| Neurolysin, mitochondrial (NLN) | 1.65 |
| Endoribonuclease ZC3H12A (ZC3H12A) | 1.63 |
| Protein LOC102724023 (LOC102724023) | 1.56 |
| MORC family CW-type zinc finger protein 3 (MORC3) | 1.53 |
| Receptor-type tyrosine-protein phosphatase F (PTPRF) | 1.51 |
| Putative ATP-dependent RNA helicase TDRD12 (TDRD12) | 1.49 |
| Isoform 2 of Very-long-chain (3R)-3-hydroxyacyl-CoA dehydratase 3 (HACD3) | 1.48 |
| Cullin-1 (CUL1) | 1.48 |
| Isoform 2 of Fizzy-related protein homolog (FZR1) | 1.47 |
| Receptor-interacting serine/threonine-protein kinase 2 (RIPK2) | 1.47 |
| Pre-mRNA-processing-splicing factor 8 (PRPF8) | 1.46 |
| E3 ubiquitin-protein ligase pellino homolog 2 (PELI2) | 1.45 |
| High mobility group protein B1 (HMBG1) | 1.44 |
| Serine/threonine-protein phosphatase 2A 65 kDa | 1.44 |
| regulatory subunit A alpha isoform (PPP2R1A) |  |
| Tetraspanin-3 (TSPAN3) | 1.43 |
| Isoform 2 of Fatty acyl-CoA reductase 2 (FAR2) | 1.39 |
| Isoform 2 of Galectin-9 (LGALS9) | 1.38 |
| Cartilage intermediate layer protein 1 (CILP) | 1.37 |
| Isoform 2 of Glutamine--fructose-6-phosphate aminotransferase (GFPT1) | 1.34 |
| Msx2-interacting protein (SPEN) | 1.33 |
| Collagen alpha-1(XII) chain (COL12A1) | 1.32 |
| 60S ribosomal protein L35 (RPL35) | 1.32 |
| Putative uncharacterized protein encoded by LINC00242 (LINC00242) | 1.31 |
| COBW domain-containing protein 3 (CBWD3) | 1.30 |
| SHC SH2 domain-binding protein 1 (SHCBP1) | 1.30 |
| Phospholipid-transporting ATPase IK (ATP8B3) | 1.29 |
| Calponin (CNN2) | 1.28 |
| Phospholipase D3 (PLD3) | 1.26 |
| PDZ and LIM domain protein 7 (PDLIM7) | 1.25 |
| Solute carrier family 12 member 9 (SLC12A9) | 1.23 |
| S-phase kinase-associated protein 1 (SKP1) | 1.23 |
| RNA-binding protein PNO1 (PNO1) | 1.22 |
| Signal recognition particle receptor subunit beta (SRPRB) | 1.21 |
| CAD protein (CAD) | 1.21 |
| Mediator of RNA polymerase II transcription subunit 24 (MED24) | 1.20 |
| Caspase-7 (CASP7) | 1.16 |
| E3 ubiquitin-protein ligase TRIM41 (TRIM41) | 1.16 |
| Mov10, Moloney leukemia virus 10, homolog (Mouse), isoform CRA_a (MOV10) | 1.15 |
| Protein SOGA1 (SOGA1) | 1.14 |
| Proteasome subunit alpha type (PSMA6) | 1.13 |
| Lon protease homolog, mitochondrial (LONP1) | 1.12 |
| Adenine phosphoribosyltransferase (APRT) | 1.12 |
| Prolactin regulatory element-binding protein (PREB) | 1.12 |
| DNA-directed RNA polymerases I, II, and III subunit RPABC3 (RPABC3) | 1.11 |
| Glutathione S-transferase Mu 5 (GSTM5) | 1.11 |
| Isoform Short of Transformer-2 protein homolog alpha (TRA2A) | 1.11 |
| Isoform 2 of Lysine-specific demethylase 5B (KDM5B) | 1.11 |
| Probable ATP-dependent RNA helicase DDX46 (DDX46) | 1.10 |
| Isoform 3 of Integral membrane protein 2C (ITM2C) | 1.10 |
| Thioredoxin-interacting protein (TXNIP) | 1.09 |
| Isoform 2 of Flotillin-1 (FLOT1) | 1.09 |
| Sideroflexin-1 (SFXN1) | 1.08 |
| Isoform 2 of DnaJ homolog subfamily A member 1 (DNAJA1) | 1.08 |
| Transforming acidic coiled-coil-containing protein 3 (TACC3) | 1.08 |
| Isoform 3 of Transmembrane protein 132B (TMEM132B) | 1.07 |
| Dolichol-phosphate mannosyltransferase subunit 1 (DPM1) | 1.06 |
| Chromodomain-helicase-DNA-binding protein 5 (CHD5) | 1.04 |
| Carbonyl reductase [NADPH] 1 (CBR1) | 1.04 |
| Nuclear pore glycoprotein p62 (NUP62) | 1.03 |
| Isoform 2 of Ras-related protein Rab-2A (RAB2A) | 1.02 |
| COMM domain-containing protein 3 (COMMD3) | 1.02 |
| Isoform Short of Long-chain-fatty-acid--CoA ligase 4 (ACSL4) | 1.00 |

**Supplementary Table 3**

Proteins found to be differentially ubiquitinated in TUBES analysis comparing control and ataxin-3 depleted THP-1 cells following MDP+PAM_3_CSK_4_ stimulation

| **Protein (Gene)** | **Log2 fold change** |
| --- | --- |
| Lysosomal alpha-glucosidase (GAA) | 3.46 |
| Rootletin (CROCC) | 2.71 |
| Adenylate cyclase type 2 (ADCY2) | 2.62 |
| DNA-directed RNA polymerase III subunit RPC1 (POLR3A) | 2.49 |
| Serine/threonine-protein phosphatase 6 regulatory subunit 1 (PPP6R1) | 2.34 |
| Serine/threonine-protein phosphatase 2A 65 kDa | 2.26 |
| regulatory subunit A alpha isoform (PPP2R1A) |  |
| Lysophosphatidylcholine acyltransferase 1 (LPCAT1) | 2.23 |
| Signal transducer and activator of transcription (STAT3) | 2.20 |
| Isoform 2 of Cardiotrophin-like cytokine factor 1 (CLCF1) | 2.19 |
| Caspase-7 (CASP7) | 2.02 |
| Isoform 2 of Ubiquitin conjugation factor E4 A (UBE4A) | 2.00 |
| Intraflagellar transport protein 74 homolog (IFT74) | 1.99 |
| Isoform 2 of Fatty acyl-CoA reductase 2 (FAR2) | 1.98 |
| Prolactin regulatory element-binding protein (PREB) | 1.97 |
| Isoform 1 of Protein POF1B (POF1B) | 1.96 |
| Poly [ADP-ribose] polymerase (TNKS) | 1.94 |
| Double-strand break repair protein MRE11A (MRE11A) | 1.84 |
| TFIIH basal transcription factor complex helicase XPB subunit (ERCC3) | 1.82 |
| Syndecan-2 (SDC2) | 1.80 |
| Isoform 2 of Cyclin-dependent kinase 2 (CDK2) | 1.79 |
| NADH dehydrogenase [ubiquinone] iron-sulfur protein 5 (NDUFS5) | 1.71 |
| Cyclin-A2 (CCNA2) | 1.69 |
| Isoform Beta-1 of DNA topoisomerase 2-beta (TOP2B) | 1.64 |
| RNA-binding protein PNO1 (PNO1) | 1.56 |
| Isoform 2 of Flotillin-1 (FLOT1) | 1.54 |
| Isoform 2 of Ras-related protein Rab-5C (RAB5C) | 1.53 |
| Ataxin-10 OS=Homo sapiens (ATXN10) | 1.48 |
| N-lysine methyltransferase KMT5A (KMT5A) | 1.48 |
| Isoform 2 of Zinc finger CCCH-type antiviral protein 1 (ZC3HAV1) | 1.48 |
| Phospholipase D3 (PLD3) | 1.43 |
| Putative sodium-coupled neutral amino acid transporter 10 (SLC38A10) | 1.43 |
| E3 ubiquitin-protein ligase ARIH1 (ARIH1) | 1.42 |
| Methionine--tRNA ligase, cytoplasmic (MARS) | 1.41 |
| Protein S100-A9 (S100A9) | 1.38 |
| Protein S100-A8 (S100A8) | 1.38 |
| E3 ubiquitin-protein ligase TRIM41 (TRIM41) | 1.37 |
| Myeloid cell nuclear differentiation antigen (MDA) | 1.36 |
| Isoform 2 of Glutamine--fructose-6-phosphate aminotransferase [isomerizing] 1 (GFPT1) | 1.33 |
| Isoform 2 of E3 SUMO-protein ligase ZNF451 (ZNF451) | 1.32 |
| Isoform 2 of Plasminogen activator inhibitor 1 RNA-binding protein (SERBP1) | 1.32 |
| Isoform 2 of Oxysterol-binding protein-related protein 5 (OSBPL5) | 1.30 |
| Isoform 2 of Solute carrier family 12 member 2 (SLC12A2) | 1.30 |
| Isoform 2 of Bifunctional UDP-N-acetylglucosamine | 1.29 |
| 2-epimerase/N-acetylmannosamine kinase (GNE) |  |
| Protein SOGA1 (SOGA1) | 1.28 |
| Chromodomain-helicase-DNA-binding protein 5 (CHD5) | 1.27 |
| BolA-like protein 2 (BOLA2) | 1.26 |
| Elongation of very long chain fatty acids protein 1 (ELOV1) | 1.26 |
| Chromodomain-helicase-DNA-binding protein 4 (CHD4) | 1.25 |
| Isoform 2 of Ubiquitin carboxyl-terminal hydrolase 48 (USP48) | 1.24 |
| Golgin-45 (BLZF1) | 1.23 |
| Isoform Short of Insulin receptor (INSR) | 1.22 |
| Isoform USP25b of Ubiquitin carboxyl-terminal hydrolase 25 (USP25) | 1.20 |
| Monocarboxylate transporter 4 (SLC16A3) | 1.19 |
| Isoform 3 of Pre-mRNA 3'-end-processing factor FIP1 (FIP1L1) | 1.18 |
| Farnesyltransferase, CAAX box, alpha, isoform CRA_c (FNTA) | 1.18 |
| Isoform 2 of Inosine-5'-monophosphate dehydrogenase 1 (IMPDH1) | 1.16 |
| Isoform 2 of ATP-citrate synthase (ACLY) | 1.15 |
| Branched-chain-amino-acid aminotransferase, cytosolic (BCAT1) | 1.15 |
| Succinyl-CoA:3-ketoacid coenzyme A transferase 1, mitochondrial (OXCT1) | 1.15 |
| Isoform 2 of Adenosylhomocysteinase 2 (AHCYL1) | 1.15 |
| Nuclear cap-binding protein subunit 1 (NCBP1) | 1.14 |
| ATP-dependent 6-phosphofructokinase, liver type (PFKL) | 1.13 |
| Krueppel-like factor 10 (KLF10) | 1.12 |
| Lys-63-specific deubiquitinase BRCC36 (BRCC3) | 1.11 |
| Isoform 2 of E3 ubiquitin-protein ligase MYCBP2 (MYCBP2) | 1.11 |
| Protein S100-A7 (S100A7) | 1.10 |
| Centrin-3 (CETN3) | 1.09 |
| Insulin-degrading enzyme (IDE) | 1.09 |
| 40S ribosomal protein S13 (RPS13) | 1.09 |
| DDB1- and CUL4-associated factor 7 (DCAF7) | 1.09 |
| Eukaryotic translation initiation factor 4 gamma 2 (EIF4G2) | 1.08 |
| Mov10, Moloney leukemia virus 10, homolog (Mouse), isoform CRA_a (MOV10) | 1.08 |
| Isoform 2 of Far upstream element-binding protein 3 (FUBP3) | 1.07 |
| 40S ribosomal protein S18 (RPS18) | 1.07 |
| ATP-dependent 6-phosphofructokinase, platelet type (PFKP) | 1.07 |
| Cathepsin D (CTSD) | 1.06 |
| Serine incorporator 1 (SERINC1) | 1.04 |
| Ragulator complex protein LAMTOR1 (LAMTOR1) | 1.03 |
| Endoplasmic reticulum resident protein 44 (ERP44) | 1.03 |
| Ubiquitin carboxyl-terminal hydrolase isozyme L5 (UCHL5) | 1.03 |
| Isoform 2 of Protein tweety homolog 3 (TTYH3) | 1.02 |
| Protein LOC102724023 (LOC102724023) | 1.02 |
| Torsin-1A-interacting protein 1 (TOR1AIP1) | 1.02 |
| Isoform 2B of Cytoplasmic dynein 1 intermediate chain 2 (DYNC1I2) | 1.01 |
| Basigin (BSG) | 1.01 |
| T-box brain protein 1 (TBR1) | 1.00 |
| Pericentriolar material 1 protein (PCM1) | 1.00 |
